# Subclinical Hypothyroidism in Pediatric Age: How Important Is Autoimmunity?

**DOI:** 10.7759/cureus.28507

**Published:** 2022-08-28

**Authors:** Patrícia Rosinha, Rosa Dantas, Márcia Alves, Teresa Azevedo, Isabel Inácio, Sara Esteves-Ferreira, Joana Guimarães

**Affiliations:** 1 Department of Endocrinology, Centro Hospitalar Baixo Vouga, Aveiro, PRT; 2 Department of Endocrinology, Centro Hospitalar Baixo Vouga, Porto, PRT

**Keywords:** anti-thyroid peroxidase antibodies, anti-thyroglobulin antibodies, isolated hyperthyrotropinemia, hashimoto’s thyroiditis, subclinical hypothyroidism

## Abstract

Background

The natural history of subclinical hypothyroidism (SHT) is influenced by the underlying etiology, being the most common Hashimoto's thyroiditis (HT) and isolated hyperthyrotropinemia (IH). Additionally, controversy exists surrounding the need for pharmacological treatment.

Methods

A retrospective observational study that included patients diagnosed with SHT caused by HT or IH at pediatric age, under levothyroxine therapy and with follow-up at Centro Hospitalar Baixo Vouga between January/2014 and July/2019. Patients with follow-up time <12 months or missing records were excluded. This study aims to compare clinical, analytical and echographic parameters and levothyroxine dose between patients with SHT caused by HT or IH.

Results

Sample of 39 patients with 16.5 ± 3.4 years, 22 (56.4%) females. There was a preponderance of females in the HT group and males in the IH (p=0.001). Changes in thyroid ultrasound were more prevalent in the HT group (85.7% vs 16.7%, p<0.001). The median initial and final doses of levothyroxine were higher in the HT group (p=0.016, p=0.011). There was a trend towards a higher levothyroxine discontinuation rate in the IH group (22.2% vs 4.8%, p=0.162).

Two positive and statistically significant correlations were found between the level of anti-thyroid peroxidase antibodies (TPOAbs) and both the final levothyroxine dose (ρ=0.544; p=0.004) and the final weight-adjusted levothyroxine dose (ρ=0.434; p=0.027).

Conclusions

HT was more common in females and was associated with higher levothyroxine requirements and less likelihood of treatment discontinuation, especially if high TPOAbs levels. These results can be useful in the difficult daily decision of starting therapy, especially in milder forms of SHT.

## Introduction

Subclinical hypothyroidism (SHT) is a relatively common condition in the general population that can manifest itself through non-specific symptoms, which has led to a less discriminated measurement of thyroid function over the years, particularly in a routine setting [1,2]. The pattern of SHT is characterized by a thyroid-stimulating hormone (TSH) value above the upper limit of the reference range in the presence of normal free triiodothyronine (FT3) and free thyroxine (FT4) levels [3]. The estimated prevalence of SHT in adults is 4-20% and in pediatric age seems to be less than 2%, despite the scarcity of epidemiologic studies in this age group [4,5].

The natural history of SHT is influenced by the underlying etiology, Hashimoto’s thyroiditis (HT) is the most common cause followed by isolated hyperthyrotropinemia (IH) [6]. As most cases of SHT in the pediatric age have a benign course and knowledge about the evolution of HT and IH is still limited, controversy regarding the treatment of this condition still exists [3]. Functional abnormalities of the thyroid gland during infancy and childhood, especially in the most severe forms of the disease (TSH >10 mUI/L), can adversely affect growth and brain maturation. The benefits of treatment with levothyroxine are only well established for the overt forms of the disease, in which mental retardation, metabolic abnormalities, growth and skeletal maturation impairment might ensue. For this reason, treatment of SHT in pediatric age remains a controversial area as no consistent association has been definitively established with adverse health outcomes [7].

Additional knowledge concerning the natural history of HT and IH and possible predictors of the need for therapy maintenance in the adult age may allow a more precise definition of which cases of SHT are eligible for treatment. This study aims to compare clinical, analytical, and echographic parameters and the dose of levothyroxine used for SHT caused by HT and IH. We intend to assess if there is any correlation between the dose required for treatment and anti-thyroid antibody levels at diagnosis.

The results of this study were previously presented as a meeting abstract at the Annual Meeting of the Portuguese Society of Pediatric Endocrinology on November 11-12, 2021.

## Materials and methods

**Figure 1:**
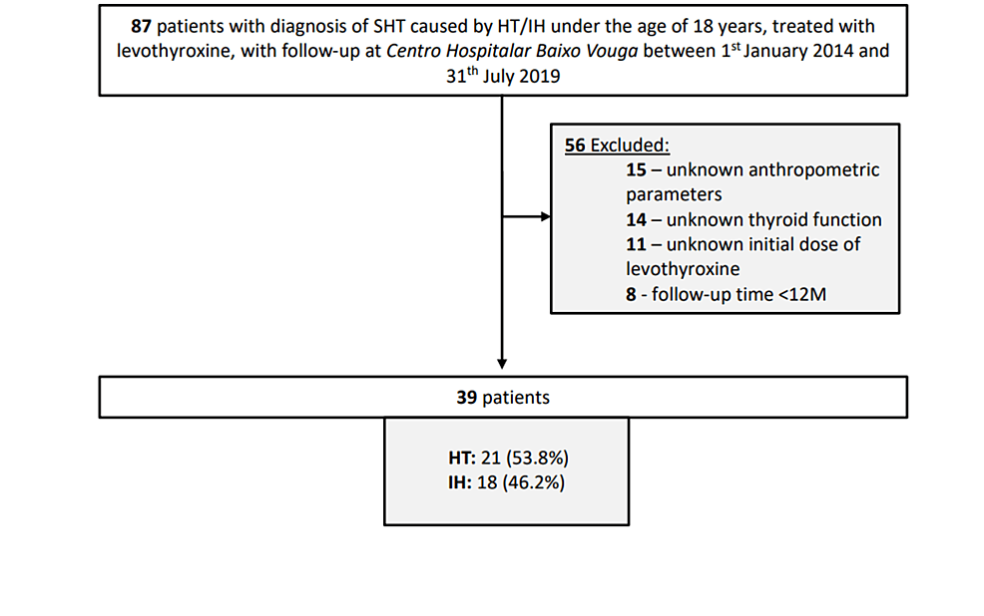
Study flowchart HT - Hashimoto thyroiditis. IH - isolated hyperthyrotropinemia.

This was a retrospective observational study including patients with SHT caused by HT or IH diagnosed under the age of 18 years, treated with levothyroxine and with follow-up in a Pediatric Endocrinology Consultation at Centro Hospitalar Baixo Vouga between January 1, 2014 and July 31, 2019. Exclusion criteria were: unknown anthropometric parameters or thyroid function at diagnosis, unknown initial dose of levothyroxine or a follow-up time of less than 12 months (Figure 1). The study protocol was in conformance with the World Medical Association’s Helsinki Declaration and was approved by the Ethics Committee of Centro Hospitalar Baixo Vouga (approval number: 39-01-2021). Informed consent was waived by the Ethics Committee based on the retrospective nature of the study and full data anonymization.

Data concerning demographic, clinical, anthropometric, analytical, ecographic and levothyroxine requirements information were collected from the clinical record. The height and weight recorded at the first visit were used to calculate the body mass index (BMI) at diagnosis as weight (kg)/height (m)^2^.

Age- and sex-specific z-scores for height, weight and BMI were calculated using the WHO AnthroPlus software based on the child growth standards from World Health Organization (WHO) [8]. Patients were categorized according to WHO classification cut-offs for BMI and height. A BMI value below -2 standard deviation (SD) was classified as underweight, ≥ -2 SD and < +1 SD as normal weight, ≥ +1 SD and < +2 SD as overweight and ≥ +2 SD as obese. Short stature was considered if height < -2 SD [9].

Serum concentrations of TSH, FT4 and FT3 were measured by ADVIA Centaur® Immunoassay Systems, considering normal ranges of 0.4-4.5 mIU/L, 0.7-1.8 ng/dL and 1.7-3.9 pg/mL, respectively. The diagnosis of SHT was made in the presence of TSH values above the upper limit of the reference range with normal FT3 and FT4 levels. SHT was classified according to the TSH value as mild if <10.0 mIU/L or severe if ≥10.0 mIU/L [7].

The presence of autoimmunity was determined by elevated serum levels of anti-thyroid antibodies (anti-thyroglobulin [TgAbs] and anti-thyroid peroxidase [TPOAbs]) and/or by the presence of an ultrasound pattern suggestive of thyroiditis (diffuse heterogenicity, pseudonodularities and/or hypoechoic micronodules). Titers of TgAbs and TPOAbs were measured by ADVIA Centaur® Immunoassay Systems and considered positive for values greater than 60 U/mL. Patients with positive anti-thyroid antibodies or an ultrasound pattern suggestive of thyroiditis were included in the HT group, while patients with the absence of these characteristic findings were included in the IH group.

Data analysis was performed using the statistical package IBM SPSS Statistics, version 20.0 (IBM Corp., Armonk, NY, USA). Categorical variables are presented as frequencies and percentages and continuous variables as means and SD. For variables with skewed distributions, medians and interquartile ranges (IQR) were presented. Normal distribution was checked using the Shapiro-Wilk test or skewness and kurtosis as appropriate. All reported p-values are two-tailed, with a p<0.05 indicating statistical significance. Categorical variables were compared with Pearson’s Chi-square test or Fisher’s exact test as appropriate. Continuous variables were compared with independent samples t-test or Mann-Whitney U-test (if skewed distribution). Spearman’s correlation coefficient (ρ) was used to assess the correlation between the dose of levothyroxine and anti-thyroid antibody levels at diagnosis. The interpretation of correlations’ strength was made using the reference values provided by Bryman and Cramer in 1995.

## Results

A total of 39 patients met the inclusion criteria for this study, with a mean age in the present analysis of 16.5 ± 3.4 years, of which 22 (56.4%) were female. The mean age at diagnosis of SHT was 11.0 ± 4.1 years, ranging between 4 and 17 years. Considering the etiology of SHT, 21 (53.8%) and 18 (46.2%) patients belonged to the HT and IH groups, respectively (Figure 1).

Table 1 shows demographic, clinical and anthropometric data by HT/IH groups. There were significant gender differences, with a preponderance of females in the HT group (81.0% vs 19.0%) and males in the IH (72.2% vs 27.8%). There were no significant differences regarding the reason for thyroid study although weight change was more frequent in the IH group (55.6% vs 23.8%; p=0.055). A total of 9 (42.9%) with HT and 5 (27.8%) in the IH group had no symptoms of thyroid disease at diagnosis (p=0.504) and no difference was found in terms of family history of thyroid disease. Anthropometric parameters did not differ significantly between groups and the prevalences of overweight, obesity and short stature were also similar (Table 1).

**Table 1:** Demographic, clinical and anthropometric data by HT/IH groups. IQR - Interquartile range. NA - not applicable. SD - Standard deviation. * Pearson's chi-square test. ^a^ - Mann-Whitney U test. ^b^ - Fisher's exact test.

	HT		IH		
	n=21		n=18		P-value
Gender - n (%)					
female	17	(81.0)	5	(27.8)	0.001*
male	4	(19.0)	13	(72.2)	
Age at diagnosis (years) - median (IQR)	12.0	(6.5)	10.0	(9.0)	0.106^a^
Age in the present analysis (years)	17.0	(4.0)	15.0	(5.0)	0.114^a^
Reason for thyroid study - n (%)					0.148^b^
weight change	5	(23.8)	10	(55.6)	0.055*
growth delay	1	(4.8)	2	(11.1)	0.586^b^
goiter/palpable nodules	3	(14.3)	1	(5.6)	0.609^b^
cognitive impairment	3	(14.3)	0		0.235^b^
other	9	(42.9)	5	(27.8)	0.504*
Symptoms of thyroid disease - n (%)	12	(57.1)	13	(72.2)	0.504*
Family history of thyroid disease - n (%)	4	(19.0)	3	(16.7)	1.000^b^
Weight at diagnosis (Kg) - median (IQR)	46.6	(31.7)	50.1	(32.7)	0.994^a^
Weight z-score - median (IQR)	0.3	(2.8)	3.2	(3.7)	0.285^a^
Height at diagnosis (cm) - median (IQR)	147.8	(27.2)	142.8	(43.8)	0.490^a^
Height z-score - median (IQR)	-0.1	(1.7)	0.9	(1.2)	0.053^a^
Height categories - n (%)					
Short stature	3	(14.3)	1	(5.6)	0.609^b^
Adequate stature	18	(85.7)	17	(94.4)	0.609^b^
BMI at diagnosis (kg/m^2^) - median (IQR)	21.3	(7.5)	20.8	(9.4)	0.791^a^
BMI z-score - median (IQR)	0.6	(2.6)	2.2	(3.8)	0.304^a^
BMI categories - n (%)					
Underweight	0		0		NA
Normal weight	12	(57.2)	8	(44.4)	0.527*
Overweight	2	(9.5)	0		0.490^b^
Obesity	7	(33.3)	10	(55.6)	0.206*

At diagnosis of SHT, the proportion of patients with mild and severe SHT was similar between groups (p=0.349) (Table 2). There was a trend towards higher TSH values in the HT group (7.64 [4.00] vs 6.26 [2.22] mUI/L, p=0.094). Median FT4 and FT3 values and time to diagnosis did not differ significantly. Thyroid ultrasound changes other than those typically found in thyroiditis were more prevalent in the HT group (85.7% vs 16.7%, p<0.001), although there were no significant differences for each one individually. In terms of levothyroxine requirements, the median initial and final doses were higher in HT group (50.0 [25.0] vs 25.0 [0.0] µg/day, p=0.016 and 75.0 [50.0] vs 50.0 [31.3] µg/day, p=0.011), although the weight-adjusted doses were similar between groups. There was no significant difference in levothyroxine discontinuation rate between groups (p=0.162) (Table 2).

**Table 2:** Analytical/echographic data and levothyroxine requirements by HT/IH groups. FT3 - free triiodothyronine. FT4 - free thyroxine. IQR - Interquartile range. NA - not applicable. SD - Standard deviation. TSH - thyroid-stimulating hormone. * Pearson's chi-square test. ^a^ - Mann-Whitney U test. ^b^ - Fisher's exact test.

	HT		IH		
	n=21		n=18		P-value
Thyroid function at diagnosis - n (%)					
mild subclinical hypothyroidism	17	(81.0)	16	(88.9)	0.349^b^
severe subclinical hypothyroidism	4	(19.0)	1	(5.6)	
Time to diagnosis (months) - median (IQR)	3.0	(3.0)	2.0	(7.0)	0.984^a^
TSH at diagnosis (mUI/L) - median (IQR)	7.64	(4.00)	6.26	(2.22)	0.094^a^
FT4 (ng/dL) - median (IQR)	0.96	(0.23)	1.10	(0.25)	0.410^a^
FT3 (pg/mL) - median (IQR)	4.20	(2.02)	3.07	(0.00)	0.952^a^
Changes in thyroid ultrasound - n (%)	18	(85.7)	3	(16.7)	<0.001*
nodules	4	(19.0)	0		0.107^b^
reactive nodes	5	(23.8)	3	(16.7)	0.702^b^
enlarged gland	6	(28.6)	1	(5.6)	0.098^b^
Initial dose of levothyroxine - median (IQR)					
µg/day	50.0	(25.0)	25.0	(0.0)	0.016^a^
µg/Kg/day	0.6	(1.1)	0.6	(0.4)	0.221^a^
Final dose of levothyroxine - median (IQR)					
µg/day	75.0	(50.0)	50.0	(31.3)	0.011^a^
µg/Kg/day	1.6	(1.7)	0.85	(1.2)	0.057^a^
Average time on levothyroxine - median (IQR)	48.0	(72.0)	43.5	(64.0)	0.994^a^
Levothyroxine discontinuation - n (%)	1	(4.8)	4	(22.2)	0.162^b^

We found two positive and statistically significant correlations: one of moderate strength between TPOAbs levels and the final dose of levothyroxine (ρ=0.544; p=0.004) and the other of weak strength between TPOAbs levels and the final weight-adjusted dose of levothyroxine (ρ=0.434; p=0.027). There was no correlation between TPOAbs levels and the initial levothyroxine dose, nor between TgAbs levels and initial/final levothyroxine dose.

## Discussion

In our study, thyroid autoimmunity was more prevalent in females and was associated with increased levothyroxine requirements both early after diagnosis and several months after disease stabilization. There was also a trend towards less likelihood of treatment discontinuation in patients with HT, although it did not reach statistical significance. On the other hand, IH tended to be associated with milder TSH elevations and the appearance of SHT at earlier ages. Thus, these results are in line with what is described in the previously existing literature, even though the number of studies on pediatric age is limited, with small sample size and high heterogeneity.

We also found correlations between TPOAbs level and both the final and the final weight-adjusted dose of levothyroxine. For many years, anti-thyroid antibodies were hypothesized to be causal agents of thyroid autoimmunity. Over the years, thyroid peroxidase (TPO) has been recognized as the main thyroid antigen and, with this knowledge, it has been suggested that part of the TPOAbs production could result from the exposure of TPO antigen to the immune system, with subsequent complement activation and potentiation of thyrocyte damage. So, TPOAbs seem to be a marker of damage to the thyroid gland and not a causative agent [10]. Therefore, children and adolescents with higher TPOAbs levels may need higher doses of levothyroxine and be less likely to discontinue therapy, in connection with more extensive damage to the thyroid gland.

We found no differences in the prevalence of overweight/obesity between groups. Previous studies have shown a positive correlation between TSH levels and anthropometric parameters, namely BMI [11]. Based on this knowledge and having found a trend toward higher TSH values in the group with autoimmune SHT, we expected to find a higher prevalence of overweight/obesity in this group, but this was not confirmed in our results. The small sample size or a more advanced pubertal stage, which might considerably reduce TSH levels, may explain this result [11]. 

Regarding the IH group, the tendency for the appearance of SHT at a younger age could be related to a genetic cause not yet identified as the underlying etiology.

This is a study of real-world data that addresses a current, relevant and controversial topic in Pediatric Endocrinology, adding information to the results of the sparse and heterogeneous studies carried out to date. However, some limitations should be noted, namely its retrospective nature which contributed to the large number of missing values found in some variables and led to the exclusion of a large number of patients, with direct repercussions on the final sample size. Furthermore, the absence of data referring to the pubertal stage might also impair the generalization of our results.

It is known that not all pediatric patients with SHT will require treatment as some will recover completely and spontaneously. The difficult part is deciding on which of them to start treatment, as we do not have markers that allow us to reliably identify the ones who benefit from it, nor do we know what is the TSH cut-off level from which levothyroxine therapy use may avoid negative outcomes. Previous studies have not been consistent with regard to the demonstrated benefits of SHT treatment in this age group, but more recently pioneering studies based on functional magnetic resonance imaging (MRI) studies that evaluated the neuronal circuits involved in memory and positron emission tomography/computed tomography (PET/CT) results over cerebral glucose metabolism suggest that, even in less severe forms of the disease, the treatment of SHT in pediatric patients may be beneficial [10].

## Conclusions

Our main findings might add some information to the subject surrounding SHT and its controversy. Actually, the natural history of the underlying etiology of SHT appears to be the cornerstone: thyroid autoimmunity is more often associated with the lifetime need for levothyroxine while its absence tends to translate into a more benign course, with only a transient need for hormonal replacement. Another key aspect to consider in the subgroup of patients with autoimmune SHT is the level of thyrocyte damage, indicated by the TPOAbs level, which was revealed to be directly proportional to the final dose of levothyroxine.

To sum up, this study shows interesting results that could be useful in the difficult daily decision of starting therapy in milder forms of SHT. Nonetheless, the small sample size makes it necessary to validate these results in large-scale studies in the pediatric population or the inclusion of several other studies of small dimensions in a meta-analysis for more valid conclusions.
